# Increasing the purity, potency and specificity of ebv-specific T cells to improve the treatment of EBV-positive lymphoma

**DOI:** 10.1186/2051-1426-3-S2-P51

**Published:** 2015-11-04

**Authors:** Sandhya Sharma, Serena K Perna, Birju Mehta, Natalia Lapteva, Rayne Rouce, Carlos Ramos, Minhtran C Ngo, Vicky Torrano, Catherine M Bollard, Ann M Leen, Adrian P Gee, Helen Heslop, Cliona M Rooney

**Affiliations:** 1 Baylor College of Medicine, Houston, TX, USA; 2 Childrens Research Institute, Houston, TX, USA

## Background

Up to ~40 % of Hodgkin and non-Hodgkin lymphomas carry the Epstein-Barr virus (EBV) genome and express type 2 viral latency proteins (T2LPs) EBNA-1, LMP-1, LMP-2 and BARF-1. EBV specific T cells (EBVSTs) can be generated from the blood of EBV+ individuals, but are usually dominated by T cells specific for viral proteins not expressed in type 2 latency lymphomas (T2LPs).

## Methods

To overcome this problem, we used dendritic cells, pulsed with overlapping peptide libraries (pepmixes) spanning T2LPs in the presence of IL-4 and IL-7 to stimulate patient peripheral blood mononuclear cells. Responder T cells were then expanded using pepmix-pulsed, autologous activated T cells and HLA-negative K562 cells expressing co-stimulatory transgenes.

## Results

Although T2LP-specific EBVSTs could be generated from healthy-donors, we were unable to consistently generate EBVSTs with significant T2LP specificity from patients. We hypothesized that patient T cells were anergic to antigen expressed within tumors, and since IL-15 has been shown to rescue tolerant or anergic T cells, we replaced IL-4 with IL-15 (in combination with IL-7). This improved antigen specificity up to 10-fold. Further dose optimization showed significant advantages in cytolytic activity, proliferation and antigen specificity with higher dose of IL-15. We achieved higher fold expansion (3 fold mean increase in absolute EBVST numbers) and enhanced specificity for T2LPs (high vs. low IL-15 concentration: EBNA1: 156±218 vs. 13±33, LMP-1: 130±223 vs. 33±68, LMP-2: 518±466 vs. 88±122 and BARF-1: 109±147 vs. 24±40; SFC/10^5^ cells; n=11). High dose IL-15 also increased the frequency of central memory T cells in the final T cell product (36.81±17 vs. 19.09±13 % vs.13.81±22 % (IL-15 Hi vs. IL-15 Lo vs. IL-4) in combination with Il-7).

EBVSTs manufactured using all three conditions were used to treat 20 patients with multiply relapsed, EBV-positive lymphoma; as adjuvant therapy after stem cell transplantation or chemotherapy in 9 patients and as treatment for disease in 11 patients. All patients in remission at the time of infusion, remain in remission. Of patients with active disease at the time of infusion, there was one stable disease among 4 patients who received IL-4/7-expanded T cells, one PR and one CR in 3 patients who received low does IL-15/7-expanded T cells and two CRs and one PR among 4 patients whose T cells were expanded in high dose IL-15 (one is too early to assess).

## Conclusions

Our results suggest that a high frequency of antigen-specific T cells in the infused product is critical for clinical activity and strategies to further improve specificity will enhance overall tumor responses.

